# Bilateral Ductal Stenting for Discontinuity of the Pulmonary Artery via the Femoral and Carotid Arteries in an Infant

**DOI:** 10.1155/2015/619653

**Published:** 2015-03-29

**Authors:** Osman Baspinar, Derya Aydin Sahin

**Affiliations:** Department of Pediatric Cardiology, Faculty of Medicine, Gaziantep University, 27310 Gaziantep, Turkey

## Abstract

Bilateral ductal stenting should be performed in cases of discontinuity of the pulmonary branches and pulmonary atresia. Performing this procedure via the carotid artery in small infants can be very difficult and challenging. We present a case of bilateral ductal stenting via both the femoral and carotid arteries in a little child with tetralogy of Fallot with pulmonary atresia and a nonconfluent pulmonary artery and bilateral ductus arteriosus.

## 1. Introduction

Bilateral patent ductus arteriosus (PDA) dependent pulmonary circulation with left and right pulmonary artery discontinuity is very rarely observed [1]. Duct dependent pulmonary circulation frequently requires a Blalock Taussig shunt or ductal stenting procedures [2]. However, discontinuous pulmonary arteries introduce technical difficulties into this process. We presented a case in which bilateral ductal stenting via the femoral and carotid arteries was performed due to tetralogy of Fallot with pulmonary atresia with the bilateral patent ductus arteriosus feeding the discontinuous bilateral pulmonary arteries.

## 2. Case Report

**Figure 1:**
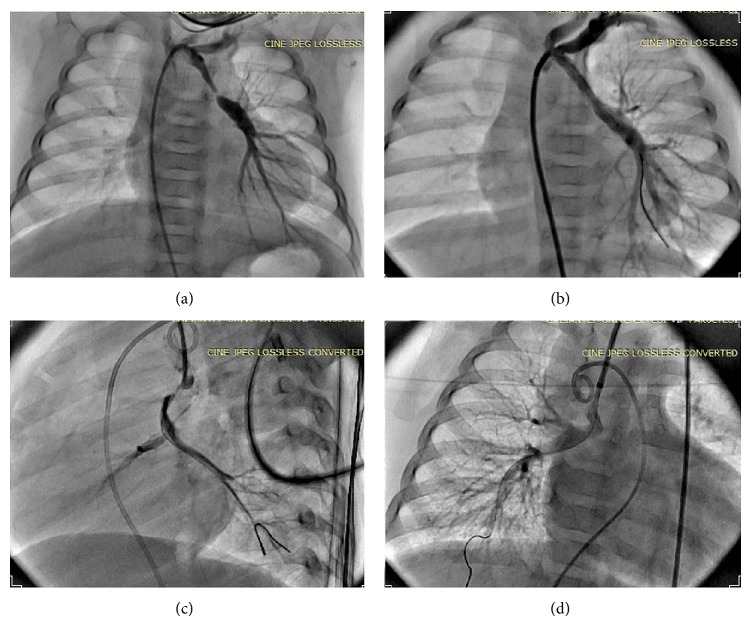
(a) Angiography via the femoral artery showing the left-sided PDA and left pulmonary artery with very severe distal ductal stenosis. (b) Angiography performed after stent placement in the left-sided PDA. Distal stenosis of the PDA has been relieved. (c) Angiography via the right carotid artery showing the right-sided tortious stenotic PDA and right pulmonary artery at the lateral projection. The right-sided PDA arises from the underside of the aortic arch and runs parallel to the descending aorta. (d) Angiography performed after stent placement in the right-sided PDA from the right carotid artery at the anteroposterior projection. The direction of the guiding catheter is straight.

A three-month-old girl with pronounced cyanosis presented to our emergency unit. Her saturation level was 40% on pulse oximetry. Transthoracic echocardiography showed that she had tetralogy of Fallot with pulmonary atresia and bilateral ductus arteriosus with nonconfluent-discontinuous pulmonary artery branches and a right-sided aortic arch. Urgent intervention with a transcatheter femoral artery approach was planned due to the surgical risk and the poor general condition of the patient. The bilateral PDAs were feeding the left and right hypoplastic pulmonary arteries separately. The left PDA, which was feeding the left pulmonary artery, had a left subclavian origin, and its diameter was decreased from 2.5 mm to 0.7 mm. During the procedure, the saturation value of the patient dropped to 25%. A 0.014-inch floppy coronary guidewire was advanced to the distal left pulmonary artery from the left PDA. A Flexor Ansel 4 Fr guiding sheath (Cook Med. Bloomington, IN, USA) was advanced through over the coronary wire. Then a 4 mm × 12 mm coronary stent was advanced to the stenotic region through the guiding sheath. Stenting of the left PDA increased to the saturation level 70% (Figures 1(a) and 1(b) and Video 1) (in Supplementary Material available online at http://dx.doi.org/10.1155/2015/619653). The right PDA originated underneath the proximal aortic arch opposite the origin of the innominate artery. The course of the right PDA was nearly parallel to that of the descending aorta, and the artery showed tortuosity and severely stenosis. After several trials, we thought that stenting the PDA via the femoral artery was impossible due to the sharp angle. The procedure was discontinued. The patient was discharged, and the patient did not come back for follow-up. Thirteen months later, the patient was referred to our emergency department again with approximately 30% oxygen saturation. She was 9 kg at this time. At the second catheterization, the left PDA stent showed in-stent restenosis, and the coronary stent was dilated a noncompliant coronary balloon. The stenosis was resolved, and the saturation level was increased to 70%. The right carotid artery was considered the most suitable approach for the right-sided PDA. The right carotid artery was punctured with a 21 gauge needle. A Flexor Ansel 4 Fr guiding sheath was inserted into the right carotid artery, via the Seldinger technique, directly from the aortic arch through the right PDA to the right pulmonary artery using a 0.014-inch floppy coronary guide wire. The right PDA diameter was decreased from 2.5 mm to 0.9 mm. A coronary stent measuring 4 × 9 mm was advanced and inflated (Figures 1(c) and 1(d), Video 1). At the end of the procedure, the saturation level was increased to 85%. Hemostasis was achieved with long-term pressure to the puncture sites. The patient was discharged two days later with an antiplatelet dose of aspirin. Total correction with conduit replacement was planned.

## 3. Discussion

Persistence of distal parts of the left and right sixth aortic arch causes bilateral PDA. It is very rarely observed and more frequently associated with nonconfluent-discontinuous pulmonary artery branches [3]. Spontaneous closure of one of the two ducts often occurs over time resulting in one functional pulmonary artery. In patients with a left-sided aortic arch, the absence of the left pulmonary artery occurs due to the closure of the left-sided PDA at the discontinuity of the pulmonary branches [2].

A ductal stenting procedure can be successfully applied as alternative to shunt surgery. Additionally, the bilateral PDA and discontinuous pulmonary artery branches require a bilateral stenting procedure [1, 2]. Double stenting increases the risk of the procedure. In a ductus with a parallel descending aorta, as in our patient, the carotid artery approach allows for the use of a more linear path to the right PDA, which is a good alternative to the femoral artery. The carotid artery approach can be used for critical aortic stenosis, coarctation of the aorta, and especially for ductal stenting. The neurological risk can be handed with good technique [4]. The advantages of the carotid approach include that it may be less harmful than femoral approaches in small children, it gives a more direct way to target the lesion, and it also provides a more stable wire route for the invasive procedure. The carotid artery approach is possible with surgical cut-down or the needle puncture technique. Surgical cut-down requires an experienced surgeon, reduced vascular complications, and freedom from compression. However, the needle puncture technique is less traumatic, and the procedural time is reduced with this technique. Therefore, we preferred the needle technique, which showed success in our cases.

The bilateral stenting procedure may make surgical repair more challenging, but not impossible, for the next corrective surgery to introduce a conduit. Therefore, we choose relatively short segment coronary stents. In addition, both procedures were performed in an urgent clinical situation.

In conclusion, ductal stenting of the bilateral PDA can be applied with success, as in our patient, using different approaches. Use of the carotid artery seems to be a logical alternative when performing difficult PDAs.

## Supplementary Material

Video 1: Angiography via the femoral artery shows the left-sided PDA and stenting procedures of it. Also, the other angiography via the carotid artery shows the right-sided PDA and stenting procedures of it.
